# Socioeconomic Disadvantage and Care Proximity Influence Stage at Presentation in Head and Neck Cancer

**DOI:** 10.1002/ohn.70261

**Published:** 2026-05-22

**Authors:** Kelly Bridgham, Praneet Kaki, Bryce Hambach, Annie Moroco, Eric Mastrolonardo, Meghan Crippen, David Cognetti, Arielle Thal

**Affiliations:** ^1^ Department of Otolaryngology–Head and Neck Surgery, Sidney Kimmel Medical College/Sidney Kimmel Comprehensive Cancer Center Thomas Jefferson University Hospital

**Keywords:** head and neck cancer, health care disparities, geographic location, neighborhood deprivation index, stage of presentation

## Abstract

**Objective:**

To evaluate how patient circumstances that may influence access to care, including area deprivation index (ADI), distance to care center, and rural/urban status affect stage of presentation for HNSCC.

**Study Design:**

Retrospective, single‐institution study.

**Setting:**

Urban, academic tertiary care center.

**Methods:**

Patients with a history of HNSCC of the oral cavity, oropharynx, hypopharynx, larynx who presented to our institution between 2018 and 2023 were included. Demographic factors including age, race, sex, ADI, insurance status, distance to tertiary care center, and rural‐urban continuum codes were collected. Primary outcome of interest was clinical stage at presentation.

**Results:**

1170 patients met inclusion criteria, of which 675 (57.7%) presented with early‐stage disease and 495 (42.3%) presented with late‐stage disease. On multivariable analysis, closer proximity to care center, insurance status, current or former smoking status, p16 negativity, and tumor subsite of the oral cavity or hypopharynx were associated with late‐stage diagnosis. After sub‐stratification for patients within the closest distance quartile, female sex, p16 negativity, non‐white and non‐black race, and insurance status remained associated with late‐stage disease presentation.

**Conclusion:**

Patient characteristics often associated with socioeconomic disadvantage including minority race, Medicaid, or lack of insurance were associated with a later stage of presentation for HNSCC. Individuals living within the closest distance to our tertiary care center were more likely to present with late‐stage disease, highlighting the need for improved access to care for socioeconomically disadvantaged urban populations.

Head and neck squamous cell carcinoma (HNSCC) is a complex and heterogeneous malignancy that poses a significant global health burden, with an incidence rate of 890,000 worldwide.^1^ Despite advances in diagnostic and therapeutic strategies, outcomes for patients with HNSCC remain largely dependent on the stage at diagnosis, with late‐stage presentation being associated with poorer prognosis and reduced survival.^2^ Identifying factors that influence stage at diagnosis is critical to improve early detection rates and develop targeted interventions.

Data has shown that demographic and socioeconomic factors such as sex, race, smoking and alcohol history, neighborhood deprivation index, access to care, and rural/urban residence can predict outcomes across various cancer types, including head and neck cancer (HNC).^3^, ^4^, ^5^, ^6^, ^7^, ^8^ The impact of sociodemographic factors on cancer stage at diagnosis has been explored in various cancer types including breast, lung, colorectal, cervical, and prostate.^9^, ^10^, ^11^, ^12^ These studies have suggested that certain patient populations, including ethnic minorities, those of lower socioeconomic class, and the uninsured are at greater risk of advanced‐stage cancer diagnosis and represent vulnerable groups who potentially face significant barriers to early detection and timely treatment. The specific interplay between demographic, socioeconomic, and geographic factors and stage at diagnosis in HNSCC remains relatively understudied, often with conflicting results. Further research is needed to identify at‐risk populations and their unique barriers to care. Understanding these barriers can inform targeted interventions to reduce disparities and improve cancer outcomes.

In this study, we retrospectively reviewed a large cohort of patients diagnosed with HNSCC at a single, metropolitan, tertiary care center to investigate the demographic and socioeconomic factors associated with stage at diagnosis in an urban setting. By identifying predictors of late‐stage presentation, we aim to guide strategies and interventions for addressing disparities in HNSCC care.

## Methods

### Patient Cohort

Electronic medical records (EMR) of patients who presented to our tertiary care center between 2018 and 2023 with head and neck squamous cell carcinoma (HNSCC) were reviewed. Patients were included if they had a confirmed diagnosis of HNSCC of the oropharynx, oral cavity, larynx, or hypopharynx, and available data to determine their American Joint Committee on Cancer (AJCC) 8th Edition clinical stage upon presentation. This study was approved by the Thomas Jefferson University Institutional Review Board (IRB).

### Demographics and Measures of Socioeconomic Status

Basic demographics including age, sex, race (White/Caucasian, Black/African American, and Other), and insurance status were collected. Social factors such as smoking and alcohol history (never, current, and former) were reported.

Area deprivation was measured using the Area Deprivation Index (ADI). This is a validated measure of socioeconomic neighborhood deprivation that is measured as a national percentile between 0 and 100 (higher scores indicate higher deprivation) based on factors such as poverty, housing, employment, and education.^13^, ^14^ The ADI was derived by entering a patient's home zip code into a publicly available website sponsored by the Center for Health Disparities Research at the University of Wisconsin School of Medicine and Public Health.^15^ Distance to the tertiary care center was calculated as the distance (in miles) between the patient's home zip code and the zip code of our tertiary care center. ADI and distance to our tertiary care center were stratified into quartiles within this patient population. Rural‐urban continuum codes (RUCC) from the US Department of Agriculture were used to characterize the rural or urban composition of the residential county for each patient based on home zip code.^16^ The RUCC categories are defined as follows: (1) counties in metro areas of 1 million population or more, (2) counties in metro areas of 250,000 population to 1 million population, (3) counties in metro areas of fewer than 250,000 population, (4) urban population of 20,000 or more, adjacent to a metro area, (5) urban population of 20,000 or more, not adjacent to a metro area, (6) urban population of 2500 to 19,999, adjacent to a metro area, (7) urban population of 2500 to 19,999, not adjacent to a metro area, (8) completely rural or less than 2500 urban population, adjacent to a metro area and (9) completely rural or less than 2500 urban population, not adjacent to a metro area.^17^ RUCC was categorized into 3 groups: large metropolitan areas (RUCC 1), small metropolitan areas (RUCC 2,3), and nonmetropolitan or rural areas (RUCC 4‐9). Year of presentation was clustered into 2 groups (2018‐2019, 2020‐2023) to determine the effect of the COVID‐19 pandemic on stage of presentation.

### Outcome Variable

The primary outcome variable was HNSCC stage at diagnosis, which was grouped into 2 categories based on 8th Edition of the American Joint Committee on Cancer (AJCC) clinical Staging of Head and Neck Cancer: Early‐stage (I/II) and Late‐stage (III/IV).

### Statistical Analysis

Numerical variables were reported as mean (standard deviation) and categorical variables were reported as frequency (percent). Chi‐square and Wilcoxon rank sum tests were used to compare categorical and continuous variables, respectively, between patients who presented with early‐stage and late‐stage HNSCC. Demographic, socioeconomic, social, and clinical factors that were significantly different between the 2 cohorts on univariable analysis were subsequently included in a multivariable binary logistic regression analysis that was performed to identify independent predictors of late‐stage presentation. Based on the results of this analysis, a separate subgroup analysis was performed separately based on p16 status and for patients within the first quartile for distance to our tertiary care center. An *α* level of 0.05 was used for all tests. All statistical analyses were performed using R Studio Version 2023.03.1.

## Results

### Demographics, Socioeconomic, and Baseline Characteristics

1170 patients met inclusion criteria, of which 675 (57.7%) presented with early‐stage disease and 495 (42.3%) presented with late‐stage disease. The average age across the cohort was 65.5 years (SD = 11.4). Patients with early‐stage disease were more likely to be male (81% vs 69%, *P* < .001), White/Caucasian (89% vs 79%, *P* < .001), never smoke (38% vs 17%, *P* < .001), and never drink alcohol (72% vs 62%, *P* < .001). A greater proportion of patients with early‐stage disease had p16+ HNSCC (83% vs 22%) and disease of the oropharynx (67% vs 21%, *P* < .001). Patients presenting with late‐stage disease had a greater national ADI percentile compared to those who presented with early‐stage disease (48.6 vs 42.3, *P* < .001). Percentile quartiles of 25th, 50th, 75th, and 100th, for distance to tertiary care center were defined at 10.4, 19.9, 48.4, and 938.4 miles, respectively, and were unevenly distributed between patients with early‐stage and late‐stage disease (*P* = .007). Among the entire population, 855 (73%) patients came from large metropolitan areas, whereas 250 (21%) came from small metropolitan and 65 (5.5%) came from nonmetro or rural areas, respectively. There was no difference in RUCC distribution between those with early and late‐stage presentation. Patients with private insurance more frequently presented with early‐stage disease (N = 331, 49%) compared to those with Medicaid, Medicare, or no insurance (Table 1). There was no significant difference in the percentage of patients presenting with late‐stage disease before (2018‐2019, 42%) the COVID‐19 era versus during (2020‐2023, 42%) the COVID‐19 era.

**Table 1:** Univariate Analysis of Demographic Characteristics Between Patients With Early Stage (I/II) and Late Stage (III/IV) Presentation

Characteristic	N	Overall N = 1170^a^	Early Stage (I/II) N = 675	Late Stage (III/IV) N = 495	*P*‐value^b^
Age	1170	65.50 (11.40)	65.11 (11.28)	66.03 (11.55)	.15
Gender	1170				<.001
Female		282 (24%)	131 (19%)	151 (31%)	
Male		887 (76%)	543 (81%)	344 (69%)	
Race	1170				<.001
White		992 (85%)	600 (89%)	392 (79%)	
Black		106 (9.1%)	47 (7.0%)	59 (12%)	
Other		7.2 (6.2%)	28 (4.1%)	44 (8.9%)	
ADI (avg, SD)	1100	44.95 (23.26)	42.25 (22.84)	48.59 (23.36)	<.001
Distance	1170				.007
Q1		293 (25%)	144 (21%)	149 (30%)	
Q2		293 (25%)	179 (27%)	114 (23%)	
Q3		293 (25%)	172 (25%)	120 (24%)	
Q4		293 (25%)	180 (27%)	112 (23%)	
RUCC	1170				
1		855 (73%)	483 (72%)	372 (75%)	.2
2‐3		250 (21%)	128 (19%)	94 (19%)	
4‐9		64 (5.5%)	290 (43%)	29 (5.9%)	
Insurance	1161				
Private		496 (43%)	331 (49%)	165 (34%)	<.001
Medicare		607 (52%)	328 (49%)	279 (57%)	
Medicaid		36 (31%)	7 (1.0%)	29 (5.9%)	
Uninsured		22 (1.9%)	6 (0.9%)	16 (3.3%)	
Smoking	1167				<.001
Never		342 (29%)	256 (38%)	86 (17%)	
Current		324 (28%)	128 (19%)	196 (40%)	
Former		501 (43%)	290 (43%)	211 (43%)	
Heavy EtOH	1163				<.001
Never		786 (68%)	480 (72%)	306 (62%)	
Current		317 (27%)	170 (25%)	147 (30%)	
Former		60 (5.2%)	21 (3.1%)	39 (7.9%)	
Subsite	1170				<.001
Oral cavity		305 (26%)	99 (15%)	207 (42%)	
Oropharynx		556 (48%)	452 (67%)	104 (21%)	
Hypopharynx		63 (5.4%)	5 (0.7%)	58 (12%)	
Larynx		245 (21%)	119 (18%)	126 (25%)	
P16 status	890				<.001
Negative		364 (41%)	95 (17%)	269 (78%)	
Positive		526 (59%)	449 (83%)	77 (22%)	

^a^ Mean (SD); n (%).

^b^ Wilcoxon rank sum test; Pearson's Chi‐squared test.

### Multivariable Logistic Regression to Identify Predictors of Late‐Stage Presentation

On multivariable logistic regression, Medicaid insurance (aHR 14.7, 95% CI 4.00, 64.9), lack of insurance (aHR 5.54, 95% CI 1.37, 25.7), current smoking status (aHR 3.79, 95% CI 2.16‐6.6.77), former smoking status (aHR 2.46, 95% CI 1.45‐4.23) disease of the oral cavity (aHR 3.28, 95% CI 1.78‐6.06), and disease of the hypopharynx (aHR 22.1, 95% CI 7.59, 83.7) were associated with late‐stage diagnosis. Greater distance to tertiary care center (aHR 0.83, 95% CI 0.70, 1.00) and p16+ disease (aHR 0.14, 95% CI 0.08‐0.23) were associated with reduced odds of late‐stage disease (Table 2).

**Table 2:** Predictors of Late‐Stage Presentation on Multivariate Analysis

Characteristic	aHR	95% CI	*P*‐value
Gender			
Female	‐	‐	
Male	1.05	0.66, 1.67	.8
Race			<.001
White	‐	‐	
Black	0.88	0.43, 1.79	.7
Other	1.75	0.78, 4.04	.2
ADI (avg, SD)	1.01	1.00, 1.02	.11
Distance Quartile (increasing)	0.83	0.70, 1.00	.04
Insurance			
Private	‐	‐	
Medicaid	14.7	4.00, 64.9	<.001
Medicare	1.34	0.90, 1.99	.20
Uninsured	5.54	1.37, 25.7	.02
Smoking			
Never	‐		‐
Current	3.79	2.16, 6.77	<.001
Former	2.46	1.45, 4.23	<.001
Heavy EtOH			
Never	‐	‐	
Current	1.18	0.77, 1.81	.5
Former	2.19	0.98, 5.02	.061
Subsite			<.001
Oropharynx	‐	‐	‐
Oral Cavity	3.28	1.78, 6.06	<.001
Hypopharynx	22.2	7.59, 83.7	<.001
Larynx	1.29	0.70, 2.36	.4
P16 status			
Negative	‐		
Positive	0.14	0.08, 0.23	<.001

Abbreviations: aHR, odds ratio; CI, confidence interval.

Multivariable logistic regression was repeated separately for patients with p16‐positive and p16‐negative disease, respectively. Among patients with p16+ disease, other race (aHR 5.30, 95% CI 1.75, 16.4), higher ADI national percentile (aHR 1.01, 95% CI 1.00, 1.03), current smoking status (aHR 5.00, 95% CI 2.45,10.6), and former smoking status (aHR 2.29, 95% CI 1.17, 4.67) were associated with late‐stage diagnosis. For patients with p16‐ disease, greater distance to tertiary care center was associated with lower odds of late‐stage presentation (aHR 0.70, 95% CI 0.56, 0.87).

### Predictors of Late‐Stage Presentation for Patients Within the First Distance Quartile

Given the apparent association between closer distance to our tertiary care center and late‐stage disease, a subgroup analysis was performed to identify risk factors for late‐stage presentation among patients within the first distance quartile (Tables 3 and 4). On univariable analysis, female sex (35% vs 14%, *P* < .001), other race (15% vs 4.9%, *P* = .003), higher average ADI (57.4 vs 47.6, *P* = .001), Medicare, Medicaid, or uninsured status (*P* < .001) were associated with late‐stage disease. In contrast, p16‐positive and oropharyngeal tumors were associated with an earlier presentation (Table 3). On multivariable analysis, other race (aHR 5.68, 95% CI 1.33, 33.7) and current smoking status (aHR 3.16, 95% CI 1.10, 9.28) were associated with increased odds of late‐stage disease. Male sex (aHR 0.31, 95% CI 0.11, 0.81), p16‐positive tumors (aHR 0.10, 95% CI 0.05, 0.22), and private or Medicare insurance (aHR 0.04, 95% CI 0.00, 0.25) were, in contrast, associated with reduced odds of late‐stage disease. ADI was not significantly associated with stage of diagnosis in this cohort (Table 4). Tumor subsite was removed from the final multivariable model due to model non‐convergence and infinite confidence intervals for patients with hypopharyngeal tumors.

**Table 3:** Univariate Analysis of Demographic Characteristics Between Patients With Early Stage (I/II) and Late Stage (III/IV) Presentation for Patients Within the First Quartile for Distance to Tertiary Care Center

Characteristic	N	Overall N = 293^a^	Early Stage (I/II) N = 144	Late Stage (III/IV) N = 149	*P*‐value^b^
Age	293	65.10 (10.28)	64.69 (9.46)	65.50 (11.04)	.3
Gender	292				<.001
Female		72 (25%)	20 (14%)	52 (35%)	
Male		220 (75%)	128 (86%)	98 (65%)	
Race	293				.003
White		195 (67%)	108 (75%)	87 (58%)	
Black		69 (24%)	29 (20%)	40 (27%)	
Other		29 (9.9%)	7 (4.9%)	22 (15%)	
ADI (avg, SD)	276	52.62 (25.42)	47.62 (25.83)	57.40 (24.16)	.001
Insurance	290				<.001
Private		113 (39%)	73 (51%)	40 (27%)	
Medicare		153 (53%)	67 (47%)	86 (59%)	
Medicaid		15 (5.2%)	2 (1.4%)	13 (8.9%)	
Uninsured		9 (3.1%)	2 (1.4%)	7 (4.8%)	
Smoking	292				<.001
Never		81 (28%)	55 (38%)	26 (18%)	
Current		93 (32%)	26 (18%)	67 (45%)	
Former		118 (40%)	63 (44%)	55 (37%)	
Heavy EtOH	293				.4
Never		186 (63%)	93 (65%)	93 (62%)	
Current		80 (27%)	41 (28%)	39 (26%)	
Former		27 (9.2%)	10 (6.9%)	17 (11%)	
Subsite	293				<.001
Oral Cavity		60 (20%)	22 (15%)	38 (26%)	
Oropharynx		132 (45%)	92 (64%)	40 (27%)	
Hypopharynx		25 (8.5%)	1 (0.7%)	24 (16%)	
Larynx		76 (26%)	29 (20%)	47 (32%)	
P16 status	235				<.001
Negative		120 (51%)	24 (21%)	96 (79%)	
Positive		116 (49%)	91 (79%)	25 (21%)	

^a^ Mean (SD); n (%).

^b^ Wilcoxon rank sum test; Pearson's Chi‐squared test.

**Table 4:** Predictors of Late‐Stage Presentation on Multivariate Analysis (for Patients Within First Distance Quartile)

Characteristic	aHR	95% CI	*P*‐value
Gender			
Female	‐	‐	‐
Male	0.31	0.11, 0.81	.020
Race			
White	‐	‐	0
Black	1.34	0.55, 3.32	.5
Other	5.68	1.33, 33.7	.032
ADI (avg, SD)	1.00	0.98, 1.01	.8
Smoking			
Never	‐	‐	‐
Current	3.16	1.10, 9.28	.033
Former	1.97	0.78, 5.17	.2
Insurance			
Medicaid/Uninsured	‐	‐	‐
Private/Medicare	0.04	0.00, 0.25	.004
P16 status			
Negative	‐	‐	‐
Positive	0.10	0.05, 0.22	<.001

Abbreviations: aHR, odds ratio; CI, confidence interval.

## Discussion

To our knowledge, this study represents the largest single‐institution study to date that evaluates the role of both patient and neighborhood‐level sociodemographic factors on the initial stage of presentation of all site HNSCC. Several expected patient and disease‐related characteristics were associated with a late stage of diagnosis, as patients who were former smokers/drinkers or had oral cavity, hypopharynx and/or p16‐negative tumors presented with more advanced disease. Furthermore, sociodemographic factors including minority race, Medicaid insurance and lack of insurance, and closer travel distance were associated with late‐stage disease. Here, we add to the growing body of literature that demonstrates socioeconomic disadvantage, both at an individual and community level, is associated with late‐stage diagnosis, and consequently worse outcomes, for HNSCC.

Advanced stage at presentation is a well‐established, negative prognostic factor for HNSCC.^2^ Social disparities are widely prevalent among the HNSCC patient population and often limit access to health care from initial diagnosis through treatment.^18^ Social determinants of health can negatively impact timely cancer care, patient quality of life, and overall survival.^18^, ^19^, ^20^ Numerous studies have demonstrated that low socioeconomic status is associated with a later stage of diagnosis and worse overall survival for HNSCC.^21^, ^22^, ^23^, ^24^, ^25^, ^26^, ^27^ The definition of socioeconomic status, however, varies greatly between studies and often relies on proxies such as race, education level, and insurance status. Likewise, in our study, several factors typically associated with a vulnerable patient population, including minority race, lack of insurance, or Medicaid were associated with a late stage of diagnosis. The ADI, on the other hand, is a composite measure of neighborhood‐level disadvantage that may provide a more comprehensive approach to understanding health care disparities.^15^, ^28^ ADI captures several critical measures of socioeconomic disadvantage across the domains of income, education, employment, and housing quality associated with a given neighborhood. Few studies to date have correlated ADI with delayed HNSCC diagnosis and treatment. A recent study by Malik et al found patients with a higher ADI were more likely to present with late‐stage nasopharyngeal carcinoma and subsequently, poor local control and decreased overall survival.^21^ In contrast, although univariate analysis in our cohort showed a trend towards later stage presentation among patients with higher ADI, this association was not statistically significant in multivariable analysis, both for the overall cohort and within the closest distance quartile.

Individuals from disadvantaged neighborhoods may face substantial obstacles to care including (but not limited to) language barriers, low education level, poor health care literacy, decreased number of health care providers in their area and lack of available transportation to treating facilities. However, the ADI is inherently limited by a lack of granularity on an individual level, particularly in heterogenous urban neighborhoods where substantial variation in access to care may exist within the same neighborhood. Individual‐level factors such as race, insurance status, health care literacy, and transportation access may play a more substantial role than neighborhood‐level deprivation alone. As such, the ADI may better serve as a population‐level risk marker of socioeconomic disadvantage rather than a surrogate for individual or community‐level deprivation in a single‐institution urban setting.

There is conflicting evidence in the literature regarding the impact of geographic location, including rural/urban status and access to tertiary care centers on head and neck cancer diagnosis and treatment.^24^, ^25^, ^29^, ^30^, ^31^, ^32^ Prior research has demonstrated an increased incidence of HNSCC in urban populations, often attributed to increased tobacco and alcohol use.^33^ Despite this, multiple studies have demonstrated an association between increased travel time and greater distance to academic care centers with an increased risk of late‐stage diagnosis, treatment delay, and worse survival outcomes.^24^, ^29^, ^30^ In line with these findings, rurality has been associated with advanced‐stage diagnosis, often attributed to increased travel time and lack of access to tertiary care providers.^34^ However, a recent systematic review found conflicting evidence regarding the role of rurality and HNSCC outcomes with 2 studies demonstrating a correlation between rurality and late‐stage diagnosis, 3 studies with urban status and late‐stage diagnosis, and 3 studies with no correlation between rural/urban status and stage at diagnosis.^32^ In contrast, our results differ from the existing literature in that living within a shorter distance to our tertiary care center was associated with advanced stage at diagnosis on multivariate analysis, suggesting an increased vulnerability for urban patients directly adjacent to our center. The largely metropolitan composition of our patient population, however, limits our ability to analyze the effect of rural/urban status, and as such our results may not be generalizable to centers with a larger rural patient population.

These findings must be interpreted within the context of our distinct location in Philadelphia, Pennsylvania. Our tertiary care center is based in a major metropolitan city that faces some of the highest poverty rates (25.8%) in the country.^35^, ^36^ There are vast differences in socioeconomic status among neighborhoods, with poverty rates approaching 40% in the area directly adjacent to our care center. Subgroup analysis for patients within the closest distance to our tertiary care center showed that the average ADI was 52.6 as compared to 45.0 for the entire patient population, highlighting the degree of socioeconomic disadvantage directly adjacent to our care center. Despite the high density of tertiary care centers present in Philadelphia, decreased access to healthcare for its residents has been consistently documented. Lack of health care literacy, language barriers, housing insecurity, and limited access to transportation disproportionately affects socioeconomically disadvantaged populations within the city.^37^, ^38^, ^39^ Our results support these findings; and highlight that despite proximity to healthcare systems, many patients face insurmountable barriers to adequate cancer diagnosis and treatment.

As demonstrated by our results, barriers to cancer diagnosis and treatment often begin upstream, where systemic inequalities such as poverty, racial disparities, inadequate housing, transportation, insurance coverage, and implicit biases result in poor access to healthcare.^40^ These population‐level factors can have substantial downstream effects at the individual level, with high levels of healthcare mistrust and avoidance documented amongst disadvantaged populations.^41^ It is critical that health care systems examine the needs of their unique patient populations to identify barriers to care and develop patient‐centered approaches to alleviate the socioeconomic, environmental, cultural, and physical barriers to care amongst their most at‐risk populations. A wide range of programs and interventions to improve access to cancer care have been evaluated. A recent systematic review identified 7 main types of interventions to mitigate barriers to cancer care, including patient navigation, education, virtual health, service‐redesign, financial support and improving geographic accessibility.^42^ Their results suggest that several of these interventions may improve access to care, particularly at the screening and diagnosis stage. However, only 1 study evaluated interventions for head and neck cancer patients specifically. Most research on improving access to care for HNSCC has focused on screening and early detection programs. A 2023 systematic review of HNC screening programs found evidence of improved survival for oral cavity and nasopharyngeal cancer^43^; however, most studies were based in Asia, where disease prevalence is high. Results of screening programs for HNC in the US are mixed, where knowledge about HNC and its associated risk factors amongst the general population are low.^44^ A retrospective cohort study of an annual HNC screening clinic over 10 years found that only 2.5% of individuals presented with findings suspicious for malignancy, and 0.2% had a diagnosis confirmed on biopsy.^45^ Further, only 0.7% of their participants fell below the poverty line. Recent focus has shifted to screening in high‐risk populations such as those identified in our study, where late‐stage presentation is more common. Studies have shown that community‐based recruitment efforts, rather than hospital‐based, are more successful at recruiting patients of low socioeconomic status and those with significant smoking and/or drinking history.^46^, ^47^ However, poor follow‐up is consistently documented amongst participants, particularly those in the higher‐risk category.^47^ Overall, community‐based screening approaches may be more effective at early detection and diagnosis for high‐risk populations, however, further efforts are needed to identify and eliminate barriers to subsequent follow‐up.

There are limitations to this study. First, there are limitations inherent to the retrospective nature including selection bias, data availability, and inability to control all confounding variables. Further, our results are limited to a single academic institution which may be impacted by regional competition and may not be representative of patients presenting to other institutions. The largely urban population of our study also may limit the generalizability of our results to other non‐urban centers. However, our results provide important insight into how sociodemographic factors influence the stage of presentation for HNSCC in an urban setting.

## Conclusion

At our urban‐based academic center, patient‐related sociodemographic factors and proximity to our care center influenced the stage of presentation for HNSCC. It is critical to understand disparities unique to a given health care system to develop targeted interventions. Community‐based screenings and educational events in high‐risk populations may help improve outreach, however, further research is necessary to improve subsequent health care follow up, timely diagnosis, and cancer treatment.

## Author Contributions

**Kelly Bridgham**, concept/design, data acquisition, data analysis and interpretation, manuscript preparation and approval; **Praneet Kaki**, data acquisition, data analysis and interpretation, manuscript preparation; **Annie Moroco**, concept/design, data analysis and interpretation, manuscript preparation and approval; **Bryce Hambach**, data acquisition, data analysis and interpretation, manuscript preparation; **Eric Mastrolonardo**, concept/design, data analysis and interpretation, manuscript preparation and approval; **David Cognetti**, concept/design, data analysis and interpretation, manuscript preparation and approval; **Meghan Crippen**, concept/design, data analysis and interpretation, manuscript preparation and approval; **Arielle Thal**, concept/design, data analysis and interpretation, manuscript preparation and approval.

## Disclosures

### Competing interests

None.

### Funding source

None.
